# Swarm SLAM: Challenges and Perspectives

**DOI:** 10.3389/frobt.2021.618268

**Published:** 2021-03-17

**Authors:** Miquel Kegeleirs, Giorgio Grisetti, Mauro Birattari

**Affiliations:** ^1^ IRIDIA, Université libre de Bruxelles, Brussels, Belgium; ^2^ DIAG, Sapienza Università di Roma, Rome, Italy

**Keywords:** swarm robotics, SLAM, distributed systems, mapping, exploration schemes, localization

## Abstract

A robot swarm is a decentralized system characterized by locality of sensing and communication, self-organization, and redundancy. These characteristics allow robot swarms to achieve scalability, flexibility and fault tolerance, properties that are especially valuable in the context of simultaneous localization and mapping (SLAM), specifically in unknown environments that evolve over time. So far, research in SLAM has mainly focused on single- and centralized multi-robot systems—i.e., non-swarm systems. While these systems can produce accurate maps, they are typically not scalable, cannot easily adapt to unexpected changes in the environment, and are prone to failure in hostile environments. Swarm SLAM is a promising approach to SLAM as it could leverage the decentralized nature of a robot swarm and achieve scalable, flexible and fault-tolerant exploration and mapping. However, at the moment of writing, swarm SLAM is a rather novel idea and the field lacks definitions, frameworks, and results. In this work, we present the concept of swarm SLAM and its constraints, both from a technical and an economical point of view. In particular, we highlight the main challenges of swarm SLAM for gathering, sharing, and retrieving information. We also discuss the strengths and weaknesses of this approach against traditional multi-robot SLAM. We believe that swarm SLAM will be particularly useful to produce abstract maps such as topological or simple semantic maps and to operate under time or cost constraints.

## 1 Introduction

A robot swarm is a decentralized system that can collectively accomplish missions that a single robot could not accomplish alone. Locality of sensing and communication, self-organization, and redundancy enable desirable properties such as scalability, flexibility, and fault tolerance (Brambilla et al., 2013; Dorigo et al., 2014) that make a robot swarm the ideal candidate to perform missions in large unknown environments in which the risk that individual robots fail or are lost is high. In particular, a robot swarm could autonomously perform simultaneous localization and mapping (SLAM) by using self-organized exploration schemes to navigate in hazardous dynamic environments. Yet, no well-defined methodology exists for performing SLAM with a robot swarm.

SLAM has been largely studied (Durrant-Whyte and Bailey, 2006) and most of the existing methods are generic, platform- and application-independent. They have been developed mostly for single robots that are usually heavily equipped and expensive. This implies that any hardware failure seriously affects the whole system. Also, they cannot be directly adapted to centralized multi-robot systems, even less to robot swarms as they usually require external infrastructures to ensure inter-robot communication or localization (a single point of failure that hinders fault tolerance).

Some important questions need to be addressed before effective swarm SLAM can be achieved: How should the swarm explore the environment and gather information? How should the robots share the information gathered? How should the information be retrieved and used to produce maps?

## 2 Literature Review

Mapping consists in creating a representation of the environment based on known robots poses and sensors data. Nowadays, it is frequently hypothesized that poses are a priori unknown and need to be estimated. Hence, the SLAM problem has been studied extensively in the past decades (Parker, 2000; Durrant-Whyte and Bailey, 2006; Dissanayake et al., 2011). A large number of methods have been developed through the years:

• for producing different types of maps—mostly occupancy grids (Elfes, 1989), but also topological (Fraundorfer et al., 2007) and semantic maps (Wolf and Sukhatme, 2008);
• to operate in generic environments (Thrun, 1998; Bailey, 2002), but also in specific ones such as underwater (White et al., 2010) or highly populated regions (Hähnel et al., 2003b);
• and using a wide variety of sensors such as cameras, LIDARs, and sonars (Elfes, 1987; Hähnel et al., 2003a; Kelly and Sukhatme, 2011).

Popular methods include GMapping (Grisetti et al., 2007; Grisetti et al., 2005), HectorSLAM (Kohlbrecher and Meyer, 2012), and KartoSLAM (Gerkey, 2014), as they are widely used in ROS (Madhira et al., 2017). SLAM was originally developed for single-robot systems and its adaptation to multi-robot systems is a more recent research direction. Mapping with multi-robot systems has been addressed in the form of two sub-problems: multi-robot SLAM (Thrun et al., 2000) and multi-robot exploration (Senthilkumar and Bharadwaj, 2012).

Multi-robot SLAM concerns the collective production of maps and estimation of robots’ position. Saeedi et al. (2016) provided a review of the many methods—based on the Extended Kalman Filter (EKF-SLAM), particle filters (PF-SLAM), and map merging, among others—that have been proposed. The review enumerates ten open issues related to multi-robot SLAM (e.g., uncertainty on robots’ relative poses, loop closure detection, out-of-sequence measurements, etc.) and evaluates widely used methods against these issues. Most of these methods are only able to address satisfactorily one or two issues, the maximum being four. A number of challenges remain: in particular, scaling the number of robots and the environment size or operating in dynamic scenarios.

Multi-robot exploration concerns the collective exploration of the environment. Despite the importance of exploration in SLAM, this task has been directly addressed more rarely than mapping and localization. Indeed, most multi-robot SLAM methods rely on path planning rather than exploration schemes specifically designed for multi-robot systems (Rone and Ben-Tzvi, 2013).

Multi-robot SLAM is still a growing field, and a number of research directions are yet to be explored. Among them, swarm SLAM is an alternative, promising approach that takes advantage of the characteristics of robot swarms. Although existing SLAM methods could be implemented in robot swarms, they would introduce constraints that would affect the flexibility and the fault tolerance of the system: centralized mechanisms or complex inter-robot interactions. The issues that one can encounter when adapting SLAM to robot swarms are described by Barca and Sekercioglu (2013). Mapping is one of the research issues mentioned by Mohan and Ponnambalam (2009) in their swarm robotics review but the authors do not elaborate on it. A rather simple swarm SLAM demonstration and a distributed localization algorithm have been reported by Rothermich et al. (2004). However, the authors do not explain how the individual maps are merged nor the role of the designer in the definition of the robots’ behavior. Moreover, the method was not properly evaluated and some information about the experimental conditions is missing, e.g., the duration of the experiments. It is only recently that Ramachandran et al. (2020) performed a real swarm SLAM experiment, which evaluates the efficiency of the so-called Informed Correlated Lévy Walk—i.e., a variant of random walk. Kegeleirs et al. (2019) performed another swarm SLAM experiment. A swarm of 10 e-puck robots had to map different bounded indoor environments using different exploration schemes. Individual maps were produced by each e-puck using GMapping and were merged afterward on a remote computer. This current limitation of the approach prevents the realization of a fully decentralized method.

## 3 Swarm SLAM

A robot swarm presents characteristics that differentiate it from centralized multi-robot systems.

First, robots in a swarm only interact with close peers and the neighboring environment. Contrary to most centralized multi-robot systems, they do not need global knowledge nor supervision to operate. Hence, modifying the size of the swarm does not require reprogramming the individual robots nor have major impact on the qualitative collective behavior. This allows robot swarms to achieve scalability—i.e., preserving performances as more agents join the system—as they can cope with any size of environment, within a reasonably large range. However, a method only working on very expensive robots will not be practically scalable in real-word application because of economical constraints that would likely prevent the acquisition of a large swarm. Hence, swarm SLAM methods should be designed taking into account the cost of the individual robots.

Then, as swarms are decentralized and self-organized, individual robots can dynamically allocate themselves to different tasks and hence meet the requirements of specific environments and operating conditions, even if these conditions evolve at operation time. This adaptation capability provides swarm SLAM with flexibility. The use of pre-existing infrastructures or sources of global information is not to be proscribed altogether, but the method should perform well regardless of the availability of these resources. For example, a pre-existing, incomplete map could be given to the robots to help them meet a critical time requirement, but the robots should be able to produce satisfactory results even if they start without any information. Flexibility is also required regarding the robotics platform: if a swarm SLAM method only works with a very specialized hardware configuration, its flexibility is compromised. Indeed, any environment or operating condition that hinder this configuration to operate would prevent the adoption of the method. Also, implementing a specialized configuration on many robots might increase the required amount of resources to an extent that would prevent any realistic large-scale application.

Finally, a robot swarm is characterized by high redundancy resulting from the large number of robots composing it. Redundancy, together with the absence of centralized control, prevents robot swarms from having a single point of failure—i.e., a component that, if unexpectedly missing or failing, prevents correct operation. Hence, a swarm SLAM method can achieve fault tolerance as the swarm can cope with the loss or failure of some robots (and also with noise, thanks to redundancy of measurements). This also requires that any equipment entirely depending on uncontrollable conditions should not be essential to succeed. For example, robots can use Wi-Fi to transmit information only if they can make use of a local communication system, should the network become unavailable. Again, fault tolerance has economical implications: losing robots should not have a significant impact on either the cost of the mission or its success. If the robots in the swarm are not expendable, a method using these robots cannot be considered fault tolerant as it could not be used in applications where losing robots is possible.

Considering these characteristics, we think that swarm SLAM is not meant to target the same applications as multi-robot SLAM: a robot swarm is most useful in cases where the main constraint is time or cost rather than high precision. Hence, they seem best suited to produce rough abstract maps, such as topological or simple semantic maps, rather than precise metric maps. Indeed, when a precise map is required, one usually has sufficient time to build it: a patrolling robot has sufficient time to build a complete map of the building it is supposed to protect before beginning its protection task. On the contrary, when time (or cost) is the main constraint, it is usually acceptable to produce approximate but informative maps: robots sent to explore a disaster area and to locate survivors can quickly give to the rescuers an approximate path to the victims location. Swarm SLAM methods also seem appropriate to map hazardous dynamic environments. When the environment evolves over time, a single or a small group of robots needs time to update the map, while a sufficiently large swarm could do it very quickly. For example, the underground exploration of unknown caverns subject to landslides could benefit from the expendable nature and the coverage offered by robot swarm. Also in this case, precision is not necessarily required, as the very fact that something has changed in the environment is usually the most valuable information: a rough representation of this modification could be sufficient.

## 4 Challenges

Given the current state of the art, it is unrealistic to expect that a swarm SLAM method can perfectly satisfy all the above constraints, at least in the short term. Scalability should be assessed more often, but large-scale experiments are difficult to perform, even with inexpensive robots. Flexibility should be achieved within reasonable constraints: a method that works indoor but not outdoor is not flexible, but a method that requires chains to be added to the robots wheels to allow them to operate in the snow could still be considered flexible. Fault tolerance is still an open issue in swarm SLAM as the most common way to produce a map in a multi-robot system, map-merging, implies some sort of centralization and hence a single point of failure. Moreover, if an heterogeneous swarm (i.e., a swarm composed of different robot types) could by itself constitute a single point of failure (if one type of robots is completely lost/destroyed), fault tolerance could still be achieved if the swarm comprises sufficiently many robots of each type.

In addition, metrics for scalability, flexibility and fault tolerance should be defined in order to evaluate swarm SLAM methods. In practice, these notions depend on aspects, such as economic or scientific hypotheses, whose quantification would be either difficult or based on arbitrary decisions—e.g., is a method not working in outer space flexible enough? Therefore, the researchers should thoroughly discuss the scalability (i.e., how large the operating environment can be?), the flexibility (i.e., how compliant the system is to different operating conditions?), and the fault tolerance (i.e., how resistant the system is to failure and perturbations?) of their methods, in particular in research involving real robots. Metrics specific to the SLAM algorithm, the exploration capabilities, and swarm robotics should also be taken into account. The SLAM algorithm should be categorized by its complexity, computation time and footprint as well as its accuracy, in the form of relative pose error (RPE) and absolute trajectory error (ATE). The exploration capabilities should be evaluated in terms of completeness and time to achieve. In the case of swarm SLAM, it is expected that completeness is reached once a sufficient portion of the environment has been explored and mapped, due to the inherent probabilistic nature of robot swarms. Finally, the complexity in the design of the swarm control software as well as the communication efforts should also be taken into account. The environmental conditions, the dynamics of this environment as well as the number of robots in the swarm and their cost would be parameter of the evaluation.

Before a scalable, flexible and fault-tolerant swarm SLAM method can be achieved, some questions need to be answered.

**How should the swarm explore the environment and gather information?**

Exploration is an essential part of SLAM. Path planning is usually the adopted strategy in multi-robot SLAM, but other exploration schemes such as Frontier-based exploration or potential fields have also been studied (Rone and Ben-Tzvi, 2013). However, in swarm robotics, simpler exploration schemes are generally used, in particular random walks (Dimidov et al., 2016; Kegeleirs et al., 2019). A straightforward option would be to adapt path planning techniques to robot swarms. Yet, these techniques have been designed for centralized systems and do not take advantage of the decentralized, collective behaviors of robot swarms. We believe that a better option would be to take advantage of swarm-specific behaviors such as aggregation/dispersion and flocking. Also, when working with robot swarms, one should consider how the control software of the individual robots will be designed. Studies have shown that the automatic off-line design of robot swarm can outperform manual design (Birattari et al., 2019; Birattari et al., 2020) by building control software from simple atomic behaviors. A recent work in automatic design has also shown that exploration capabilities might come from the interaction between atomic behaviors and not only from the exploration schemes embedded in these atomic behaviors (Spaey et al., 2020). Using simple, swarm-specific exploration schemes would hence be beneficial to both the design process and the efficiency of a swarm SLAM method.

Regarding the information to be gathered, the experiment of Kegeleirs et al. (2019) has shown that a robot swarm can produce an occupancy grid of a closed indoor environment in simulation, but struggles in reality because of poor-quality close-range proximity sensors. This means that, provided with the right sensors, a robot swarm can potentially produce any kind of map, as shown by Allen et al. (2020). However, swarm SLAM methods would benefit from low-cost, simple robots that will likely have imprecize sensors. They should hence focus on more abstract maps that do not require high precision. A promising, distributed approach for building semantic maps has been proposed by Rosinol et al. (2020), even though its computational complexity might be too high for robot swarms.

Concerning localization, a distributed localization method is required, but little research exists on this subject (Roumeliotis and Bekey, 2002; Prorok et al., 2012). Nonetheless, if high precision is not a requirement, an approximation of each robot’s location is acceptable and the localization issue becomes easier to solve.

**How should the robots share the information gathered?**

When mapping with multiple robots, information must be shared at some point. The most common approaches in multi-robot SLAM are raw and processed data sharing (Saeedi et al., 2016). With a robot swarm, neither seems optimal. Sharing raw data from the sensors is straightforward, but it might scale poorly as the huge amount of data could become impossible to transfer quickly enough. Sharing processed data could solve this problem by reducing the amount of data to be shared, but most existing methods are centralized and rely on external infrastructures such as GPS or remote computers to assemble the different subsets of data.

A fault-tolerant option would be to use a mobile ad-hoc network such as the one proposed by Di Caro et al. (2005), or the distributed approach presented by Majcherczyk et al. (2020). When mapping dynamic environments, if the valuable information is only the location at which a modification happened, a very schematic map could be sufficient and drastically reduce the amount of data to be shared. A few promising candidates to achieve fully decentralized swarm SLAM are distributed mapping (Fox et al., 2006; Ghosh et al., 2020; Lajoie et al., 2020) and graph-based mapping (Kümmerle et al., 2011)—the latter seems particularly appropriate for building topological or semantic maps.

Some practical issues also need to be addressed. Reaching a consensus in a decentralized system requires additional delays and data sharing, which cannot be neglected for cost- or time-constrained applications. Yet, in swarm SLAM, this consensus is controlled by locality and effective divide-and-conquer strategies could lessen the consensus cost (Yazdani et al., 2019). Also, practical scenarios might require a more sparse distribution of the swarm that would limit inter-robot communication (Tarapore et al., 2020).

**How should the information be retrieved and used to produce maps?**

Retrieving the map without centralizing the information is an open issue in swarm SLAM. Indeed, the most intuitive approach, map-merging, requires the individual maps to be gathered on a single system to merge them, like in the experiment of Kegeleirs et al. (2019). A solution could be to merge the individual maps in all the robots and then to retrieve the map from any of them, but this is unrealistic without the use of an external infrastructure. Again, a mobile ad-hoc network could preserve the system’s fault tolerance. In this case, we believe that the amount of data transiting by each robot for sharing an occupancy grid would be too hefty in a large environment, causing important delays. It would also require significant storage capacity on each robot, increasing the general cost. However, this solution might work with abstract maps that require less data, especially when mapping dynamic environments.

Finally, one can consider a situation in which retrieving the map is not necessary. Indeed, retrieving the map mostly makes sense if a human operator needs it, either for themself or for transferring it to another robotic system. While this is often the case, as the purpose of most SLAM methods is precisely to build maps to be used by another party, one could consider maps that are only useful for the robots that built it. For example, a cleaning robot builds maps whose sole purpose is to help the robot navigate the environment. In swarm robotics, building a map could help the robots in their exploration and improve their performance. This map does not need to be accessible to the human operators and can hence be shared, completely or even partially, among the robots only.

## 5 Conclusion

In this paper, we have reviewed the current state of the art in multi-robot and swarm SLAM. Swarm SLAM is currently an emerging research topic that lacks definitions, frameworks, and results. We have presented swarm SLAM methods as alternatives to autonomously build a map in a decentralized, scalable, flexible and fault-tolerant way. This implies a number of constraints that we have discussed, both in their technical and economical implications. We have then sketched our vision of future applications of swarm SLAM as well as the main challenges in this SLAM approach. We believe that swarm SLAM could play an important role in time- or cost-constrained scenarios or for monitoring dynamic environments. However, to fulfill these goals, swarm SLAM still needs appropriate, distributed data sharing strategies, both among robots and between robots and human operators. Moreover, a thorough examination of swarm exploration schemes could benefit to both swarm SLAM and swarm robotics in general.
